# Editorial: Recent trends and spotlight on nucleotide-based drugs: novel targets, their design, delivery, and clinical potential

**DOI:** 10.3389/fphar.2023.1245809

**Published:** 2023-07-13

**Authors:** Subhash Chander, Shvetank Bhatt, Kamal Dua, Hemant Jadhav

**Affiliations:** ^1^ Amity Institute of Phytomedicine & Phytochemistry (AIP&P), Amity University Uttar Pradesh, Noida, Uttar Pradesh, India; ^2^ School of Health Sciences and Technology, Dr. Vishwanath Karad MIT World Peace University, Pune, Maharashtra, India; ^3^ Discipline of Pharmacy, Graduate School of Health, University of Technology, Sydney, NSW, Australia; ^4^ Faculty of Health, Australian Research Centre in Complementary and Integrative Medicine, University of Technology, Sydney, NSW, Australia; ^5^ Uttaranchal Institute of Pharmaceutical Sciences, Uttaranchal University, Dehradun, India; ^6^ Department of Pharmacy, Birla Institute of Technology and Science, Pilani, Rajasthan, India

**Keywords:** nucleotide, Parkinson, oligonucleotide, nasal delivery, targeted delivery, pharmacokinetic

Therapeutics comprising of nucleotide entities is very broad class, covers nucleotide analogs, oligonucleotides and nucleic acid-based based therapeutic. Nucleotide/nucleoside-based drugs are already very well explored, with clinically approved candidates as antiviral, anti-cancer, anti-bacterial and anti-rheumatoid etc (Garner, 2021). Oligonucleotide therapy is relative recent and promising, comprising of Antisense Oligonucleotides (ASOs), Small Interfering RNAs (siRNAs), Short Hairpin RNAs (shRNAs), Anti-MicroRNAs (anti-miRs). Oligonucleotides selectively bind to RNA or proteins, blocking their function or promoting degradation. Oligonucleotide therapy shows potential in treating genetic disorders, cancer, viral infections, and neurodegenerative conditions (Roberts, 2020).

Currently, 15 oligonucleotides therapeutics are approved for treatment of various rare diseases in the United States, in which four are approved against Duchenne muscular dystrophy. Particularly, in March 2020, approval of viltolarsen attracted attention of researchers worldwide towards oligonucleotides therapeutics (Igarashi, 2022).

Nucleic acid-based therapies comprising of long polynucleotides target the diseases at the genetic level, and aiming to modulate gene expression, correct genetic mutations, or interfere with disease-causing processes (Sridharan, 2016). Discovery of CRISPR-Cas9 gene editing technology has revolutionized nucleic acid-based therapeutics, however, still research enduring on improving their delivery methods, enhancing targeting specificity, and ensuring safety and efficacy of such therapeutic agents (Uddin, 2020).

Researchers are striving continue to explore the translational potential of oligonucleotides, while addressing various associated challenges like specificity, delivery, off-target effects, and undesirable pharmacokinetic profile. Numerous trials are underway, exploring their suitable route of administration, targeted delivery, optimized formulations, and long-term therapeutic effects. The translation of oligonucleotide could provide solution of various diseases in terms of precision medicine, which can treat diseases at molecular level (Moumne, 2022).

Under the current Research Topic, five review and one research, total six studies are published. First study covers the potential of oligonucleotide therapeutics (OT) emphasising on patient customization, while second review discussed on intranasal delivery of nucleic acid-based therapeutics, targeting the CNS disorders. Next two review focus on Parkinson’s disease, one cover nasal delivery of nucleic acid-based therapeutics and while another discussed miRNA and antisense oligonucleotide. Fifth review study emphasized on nano-siRNA based approaches in treatment of cancer, while research study illustrated the immunostimulatory responses of RES-010, an antimiR-22 oligonucleotide on mononuclear cells.

Study by Thakur et al. highlights the past and present of OTs, emphasizing on the ongoing efforts to develop them as future therapeutics. Study specially highlighted their potential against the targets, which are undruggable for the conventional drugs. Overview of OTs, especially antisense RNAs, microRNAs, small interfering RNAs, and aptamers, being explored for the treatment of neurodegenerative disorders, cancer, and orphan diseases etc., is provided in the study. Key safety aspects of OTs like persistent gene silencing, off-target related toxicities, immuno-stimulatory responses, Renal accumulation, thrombocytopenia, and coagulation inhibition etc., are pointed in the study. Study discussed oligonucleotide instability, delivery-related issues, briefly provide overview of delivery vector. Strategies adopted to overcome the challenges faced with oligonucleotide delivery are summarized, including advanced drug carrier systems.

Study by Shah et al., summarized the potential of nucleic acid-based therapeutics (NBTs) in treating various diseases like cardiovascular, inflammatory, neurological, hereditary disorders, cancer, and infectious diseases. Delivering NBTs to the target sites poses several challenges, study covers such challenges like stability, target specificity, biodistribution, and suitability of administration via non-invasive routes. Study illustrated nasal delivery an alternative non-invasiveness route for NBTs, with several advantages like sustained delivery potential, improved patient compliance, and ability to bypass the blood-brain barrier. Study also covered the recent approaches being undertaking like chemical modifications, delivery technologies, and nanoplatforms to troubleshoot delivery challenges.

Pandey and Singh briefly discussed pathophysiology of Parkinson’s Disease (PD), and illustrated accumulation of α-synuclein aggregates, known as Lewy bodies as hallmark pathology of disease. Elaborating the nucleic acid therapeutics, including antisense oligonucleotides, microRNA, and gene therapy against PD, review study stated their potential role in modulating genetic dysregulation and downregulating α-synuclein expression. Superiority and associated challenges of NBTs, related to target specificity, stability, toxicity, and pharmacokinetics are also covered in this study. Although, studies support positive outcome of gene therapy approaches in PD by restoring function and ameliorating symptoms, however, further research and more clinical trials are required to validate their efficacy and safety in PD patients.

Review study by Suvarna et al. focuses on recent advances in oligonucleotides based therapeutics against the Parkinson’s disease, particularly by targeting alpha-synuclein protein. Study provided an overview of alpha-synuclein, aggregation mechanism, and its significant role in neuronal dysfunction and death, particularly in Parkinson’s disease. Study covers recent advances in oligonucleotide chemistry, leading to the development of potential alpha-synuclein targeting molecules. Study discussed ASOs, suppressing the expression of the SNCA, gene responsible for alpha-synuclein production, preventing neurodegeneration. MiRNA inhibitors have shown potential in downregulating dysfunctional miRNAs involved in Parkinson’s disease. Study discussed associated challenges with ASOs and miRNA-based therapeutics as well as undergoing research to address the challenges, particularly chemical modifications approach to enhance target specificity, stability, and reduce off-target effects is well illustrated.

Study by Goyal et al. discussed various perspective of SiRNA-based therapies against cancer, starting from introduction, mechanistic aspects, safety, selectivity, and associated challenges in translating them into clinical setting. Review also discussed on the combination of siRNA with chemotherapeutic drug delivery systems for the treatment of cancer and provided an overview of several nanocarrier formulations for siRNA delivery which are currently being explored. Study reviewed scope of chemical modifications of siRNAs, and its effect on their therapeutic efficacy and stability. Study advocated simultaneous targeting of multiples genes with combination of siRNAs and chemotherapy to achieve better therapeutic outcome. SiRNA nanoparticle delivery relies on the EPR effect, but exclusive selectivity on silencing siRNA target genes in tumor tissues needs to be assessed. The development of effective delivery mechanisms and evaluation of safety and effectiveness in clinical trials are suggested as deciding factors for the advancement of siRNA-based therapies.

Research study reported by Panella et al. was an extension of work on RES-010, a chemically modified antisense oligonucleotides (ASOs). Research study briefly provide background of MicroRNA, with special emphasis on MicroRNA-22 (miR-22), later is documented crucial in maintaining lipid and energy balance, and promising target for addressing NAFLD and obesity. In this study, impact of RES-010 was evaluated for immune responses on peripheral blood mononuclear cells (PBMCs) of human origin. PBMCs obtained from six healthy volunteers were treated with various concentrations of RES-010, subsequently, levels of proinflammatory cytokines were assessed. Study evaluated T-cell activation (based upon the markers like CD69, HLA-DR, and CD25) and cell viability after exposure to RES-010. Findings of the study indicated that RES-010 did not elicit significant immunostimulatory responses in human PBMCs *in vitro* compared to control conditions, consequently, suggested its low proinflammatory potential.

In conclusion, Research Topic, entitled *Recent trends and spotlight on nucleotide-based drugs: novel targets, their design, delivery, and clinical potential*, discussed various studies published on nucleic acids-based therapeutics, especially oligonucleotides category. Published articles highlights intriguing potential of oligonucleotides therapeutics against Parkinson, Cancer, and other physiological disorders. Published studies covered the molecular pathways, targets, therapeutic potential, development status, associated challenges, and future perspectives of oligonucleotides therapeutics.
